# Cross-shelf investigation of coral reef cryptic benthic organisms reveals diversity patterns of the hidden majority

**DOI:** 10.1038/s41598-018-26332-5

**Published:** 2018-05-24

**Authors:** J. K. Pearman, M. Leray, R. Villalobos, R. J. Machida, M. L. Berumen, N. Knowlton, S. Carvalho

**Affiliations:** 1 King Abdullah University of Science and Technology (KAUST), Red Sea Research Center (RSRC), Biological and Environmental Sciences and Engineering (BESE), Thuwal, 23955-6900 Saudi Arabia; 2 Smithsonian Tropical Research Institute, Panama City, Balboa, Ancon Republic of Panama; 3 Biodiversity Research Center, Academia Sinica, Taipei, Taiwan; 4 National Museum of Natural History, Smithsonian Institution, Washington, DC USA

## Abstract

Coral reefs harbor diverse assemblages of organisms yet the majority of this diversity is hidden within the three dimensional structure of the reef and neglected using standard visual surveys. This study uses Autonomous Reef Monitoring Structures (ARMS) and amplicon sequencing methodologies, targeting mitochondrial cytochrome oxidase I and 18S rRNA genes, to investigate changes in the cryptic reef biodiversity. ARMS, deployed at 11 sites across a near- to off-shore gradient in the Red Sea were dominated by Porifera (sessile fraction), Arthropoda and Annelida (mobile fractions). The two primer sets detected different taxa lists, but patterns in community composition and structure were similar. While the microhabitat of the ARMS deployment affected the community structure, a clear cross-shelf gradient was observed for all fractions investigated. The partitioning of beta-diversity revealed that replacement (i.e. the substitution of species) made the highest contribution with richness playing a smaller role. Hence, different reef habitats across the shelf are relevant to regional diversity, as they harbor different communities, a result with clear implications for the design of Marine Protected Areas. ARMS can be vital tools to assess biodiversity patterns in the generally neglected but species-rich cryptic benthos, providing invaluable information for the management and conservation of hard-bottomed habitats over local and global scales.

## Introduction

Understanding how diversity is partitioned along natural and anthropogenic gradients within ecosystems is a central topic in ecology and a key goal for conservation^1,2^. Cross-shelf habitats present some of the sharpest gradients in physical and chemical conditions (e.g. salinity, temperature and nutrients) in marine systems^3–5^. Near-shore areas are under the influence of terrestrial systems while outer regions are more exposed to oceanic currents and wave action^3–6^. These environmental differences, in turn, affect the distribution of marine organisms as a function of taxon-specific physiological requirements and life history traits^7^. For example, Ellis and co-authors^8^ indicated that nutrient availability across the shelf gradient in the southern Red Sea was associated with patterns of macro-algal cover (higher in nutrient enriched near-shore sites) and scleractinian coral diversity (higher at off-shore sites with lower levels of nutrients). Further in the central Red Sea distinct gradients across the shelf have been detected in physio-chemical parameters (e.g. chlorophyll and sedimentation)^9^ as well as differences in the coral community with *Acriporoa and Pocillopora* being comparatively rare in near-shore reefs^10^.

On coral reefs, various studies have reported cross-shelf differences in population size, biomass, recruitment rates and species composition among fish^11–13^, corals^10,14^, sponges^15,16^, molluscs^17^, crustaceans^18^ and siphonophores^19^. However, studies assessing multiple taxonomic groups have received less attention and have predominantly focused on more conspicuous taxa^5,7,8,20–24^. As a result, there is still a poor understanding of community-wide changes both in terms of the composition and structure across inshore-offshore gradients, especially in highly diverse tropical ecosystems such as coral reefs. This is particularly true for the small and highly diverse cryptic invertebrates that exist within the reef matrix^7,25–29^. This neglect is due to the fact that many of these species are either undescribed or very difficult to identify.

Despite being overlooked in most of the coral reef research, benthic cryptic fauna include a variety of sessile and mobile taxa^30–35^ inhabiting hidden spaces that can account for up to two thirds of the reef’s volume^36^. Little is understood about the life histories and habitat preferences of these organisms beyond size-related generalizations^37^. Some studies have investigated the community composition patterns at very small spatial scales and found that crevices within reefs are often dominated by coralline algae near sunlit entrances and by filter feeders in posterior sections^38,39^. Species inhabiting these cryptic spaces, especially Porifera, act as a sink for dissolved organic matter, thus preventing energy and nutrient losses into the open ocean^40^. Therefore, a better understanding of the distributional patterns of the crypto-benthic fauna may reveal insights into biogeochemical cycles within the reef system.

In recent decades, standardized sampling units have been proposed as a means to quantify the diversity and composition of benthic assemblages in a consistent manner across space and time. In this study, we use Autonomous Reef Monitoring Structures (ARMS) which are designed to partially mimic the 3D structure of a reef^41,42^. The structures enable the study of both the sessile components growing on the plates as well as the mobile organisms that settle on or move into the spaces. The communities collected in ARMS are typically analyzed using some form of genetic barcoding (of individual organisms) (e.g.)^43^ or high-throughput amplicon sequencing (i.e. metabarcoding of communities) (e.g.)^42^. Studies utilizing ARMS have revealed much about the hidden diversity of the Indo-Pacific and Caribbean^43^, as well as using high throughput amplicon sequencing (i.e. metabarcoding) to assess the sessile and mobile fractions on the US Atlantic coast^42^ and the Red Sea^44,45^.

Cryptic organisms include a diverse selection of ecologically important groups such as suspension feeders^46,47^, predators^48^, herbivores^49^ and detritivores^50^. Because of their smaller sizes, they also have larger population sizes and faster generation times^51^. Their response to environmental changes may therefore differ from those of the better-studied fish and corals. The cryptic fauna represents a large proportion of the reef diversity, and thus to best conserve biodiversity of the reefs across spatial scales, an understanding of how the cryptic fauna responds to environmental changes is vital^33^. To achieve this, an assessment of not only the local species assemblages (alpha diversity) but also the inter-site differences (beta diversity) is required^2^. While the total number of species is an important element in conservation, the change in species across space strongly influences the optimal spatial arrangement of conservation areas^26^.

Besides a mere quantification of the differentiation of biological communities, beta diversity can be partitioned into different components allowing for a more comprehensive understanding of the mechanisms driving diversity patterns^52^. For example, Legendre^52^ partitioned beta diversity into species replacement and richness difference. Species replacement refers to the substitution of species along an environmental/temporal gradient due to environmental filtering, competition, and historical events^53^. On the other hand, species richness refers to the number of species contained within each community, which may reflect the diversity of niches available at different locations^52^. A difference in the relative contribution of these two components to total beta diversity suggests that the structure of species assemblages is being controlled by different mechanisms^54^. Understanding the contribution of these partitions is vital to linking dissimilarities amongst communities to ecological processes^55^ and subsequently to informing conservation and management decisions^54^.

Here, we assess the composition and structure of the cryptobenthic communities that colonized ARMS at eight reefs (11 sites) in the central Red Sea region, an area of high diversity and endemism^56^. We use metabarcoding sequencing of two gene regions that differ in their level of taxonomic coverage and resolution to better reflect the diversity of life forms on the ARMS^57^ as well as standard barcoding of the largest organisms. For metabarcoding, we used highly versatile PCR primers available for the slowly evolving 18S rRNA to target the whole eukaryotic domain and general primers optimized for metazoans for the hypervariable mitochondrial Cytochrome C Oxidase Subunit I (mtCOI) with general primers optimized for metazoans.

Based on trends in coral reef fauna observed within the region^8^ and globally^10,11,14,15^ we hypothesized that there would be substantial changes in the community structure and composition across the shelf gradient. Also we expected the near-shore reefs to be more depauperate in nature (i.e. lower species richness) due to a stronger influence from human activities and higher levels of sedimentation. We also hypothesized that the different faunal components would respond similarly to changes in environmental conditions because of the sharp cross-shelf environmental gradients. Finally, we also expected patterns of alpha and beta diversity obtained from the two independent target genes to be correlated despite targeting different fractions of the communities.

Overall, our analysis provides the first comprehensive assessment of the biodiversity changes across shelf gradients in cryptic reef organisms by combining information on alpha and beta diversity (partitioned into replacement and richness). Our analysis illustrates how standardized sampling approach combined with molecular techniques (i.e. PCR-based analysis of two marker genes) can provide a basis for the comparative assessment of cryptic benthic organisms in hard bottomed substrates and help understanding how anthropogenic pressures affect this functionally important component of benthic communities.

## Methods

### Environmental data

Satellite data were downloaded from the NASA Oceancolor website (https://oceancolor.gsfc.nasa.gov/ downloaded 14^th^ February 2017). Monthly averages of chlorophyll (MODIS A) and sea surface temperature (SST) (MODIS A) at a 4 km resolution were retrieved. The data value for each reef site was based on the value associated with the nearest appropriate grid point. Due to the spatial resolution of the data, some sites were assigned to the same grid point and thus had the same environmental data.

### Sampling strategy and laboratory processing

**Figure 1:**
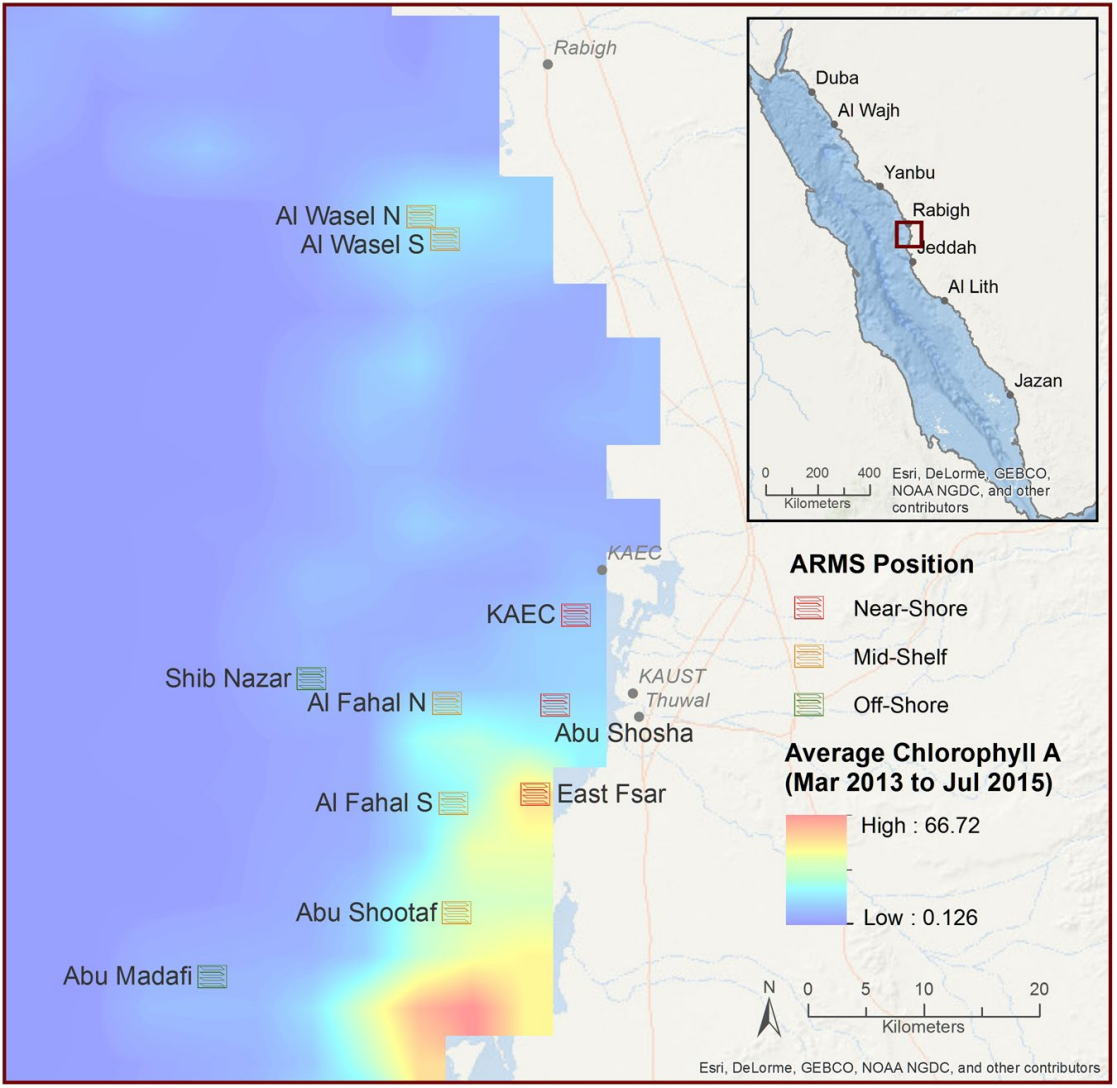
Chlorophyll *a* concentrations (mean of composite monthly averages over the time frame of the study) for the study regions derived from NASA’s Oceancolor website (https://oceancolor.gsfc.nasa.gov/) derived from the MODIS A satellites at a 4 km resolution.

**Figure 2:**
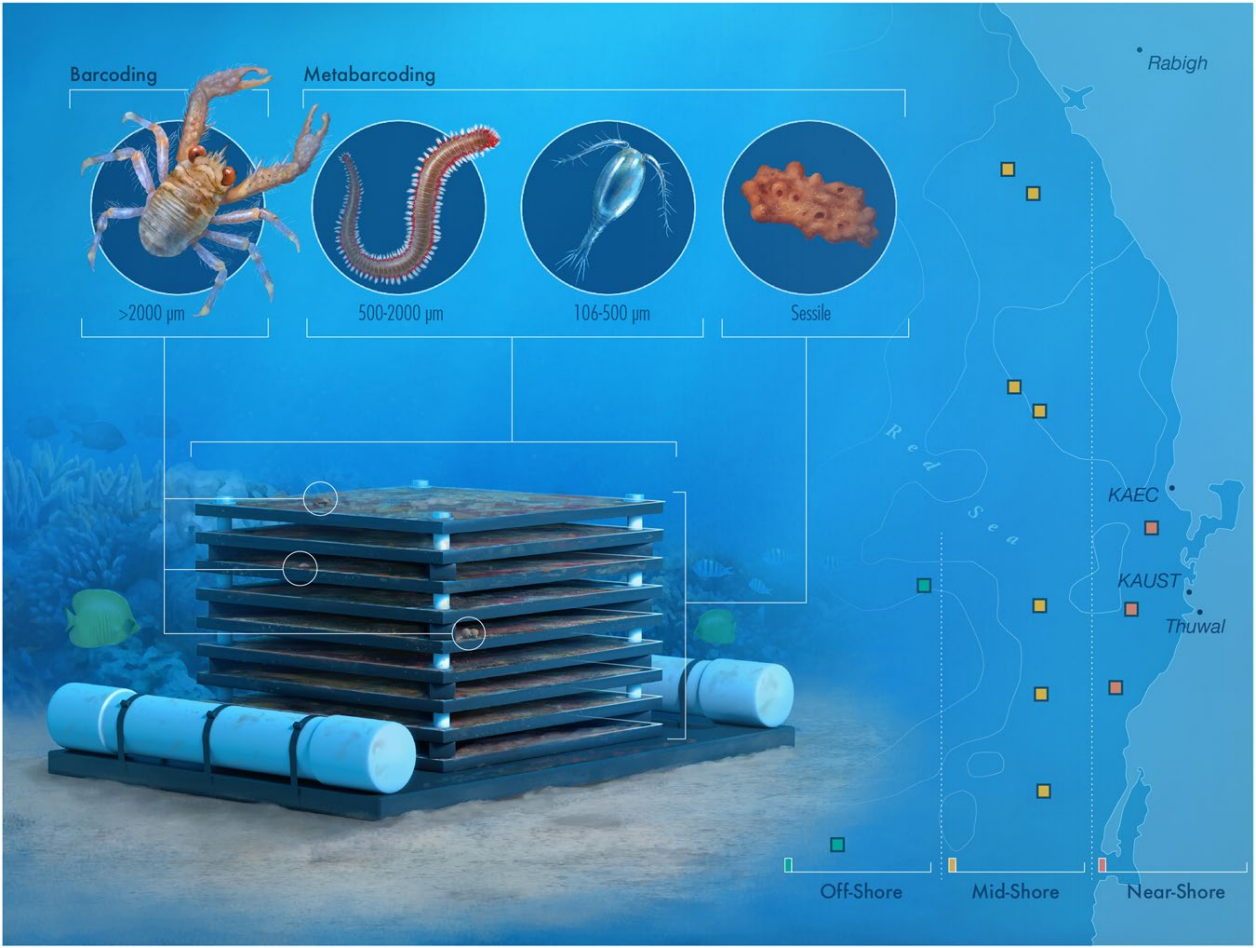
Methodological approach to ARMS sampling in the central Red Sea.

Triplicate ARMS were deployed on 11 reef sites near Thuwal (Lat: 22.30N; Lon 39.12E) in the central Red Sea for two years at approximately 10 m depth (Fig. 1 and Supplementary Table S1). The reefs were characterized as either near-shore (n = 5), mid-shelf (n = 4) or off-shore (n = 2), according to the distance to the shore (near-shore, <5 km; mid-shelf, 5–25 km; off-shore, >25 km) (Fig. 2). ARMS were deployed in two separate batches. A first deployment of four sets of three ARMS was deployed in February 2013. They were positioned on hard coral framework (later referred to as reef). A second deployment of 7 sets of three ARMS was conducted in May/June 2013. They were positioned on loose coral rubble (later referred to as rubble). The times of deployment and retrieval for each, as well as the depth for each reef site, are detailed in Supplementary Table S1. Overall, deployment, retrieval, and laboratory processing of the units, as well as the extraction of the DNA, followed the procedures previously described^42^. ARMS were retrieved so as to retain all organisms on or inside the units. These organisms were subsequently sorted into various size fractions for analysis. For the largest samples (>2000 μm) DNA was extracted for individual barcoding using the DNeasy kit (Qiagen) instead of the AutoGeneprep 965 (AutoGen). The smaller fractions (Sessile, 106–500 μm and 500–2000 μm) were processed for metabarcoding as described by^42^.

### DNA amplification and sequencing

Two primer sets were used to amplify eukaryotic sequences from the various fractions of the ARMS units (see Supplementary Table S2 for sequences). A 313 bp fragment of the mitochondrial COI gene was targeted using versatile PCR primers^58^ and PCR conditions previously described^42^. The v4 region of the 18S rRNA gene was amplified using primers designed by^59^ and using the PCR conditions described by^45^. Triplicate PCR products were merged, cleaned of primer dimers using streptavidin magnetic beads and normalized, before a second round of PCR amplification was undertaken using the Illumina 16S metagenomic sequencing library preparation protocol. An Illumina MiSeq sequencing platform (v3 chemistry) was used with the sequences (2 × 300 bp) being generated at the King Abdullah University of Science and Technology (KAUST) Bioscience Core Laboratory (BCL). Raw reads were deposited at Figshare with the following doi’s: 10.6084/m9.figshare.5559106.v1, 10.6084/m9.figshare.5558113, 10.6084/m9.figshare.5549365, 10.6084/m9.figshare.5552782, 10.6084/m9.figshare.5552932.

Extracted DNA for the barcoding samples was amplified using the mitochondrial COI gene primers jgLCO and jgHCO (Supplementary Table S2) and the PCR mix as described by^60^ with the addition of 0.2 μL Bovine Serum Albumin (BSA) (20 mg/mL) and using the PCR thermal cycling conditions detailed previously^42^. For specimens for which amplification could not be obtained using the jgLCO/jgHCO primer combination, PCRs were repeated using the dgLCO/dgHCO primer set with the conditions as described by^61^. Primers were cleaned and sequenced on either a Sanger ABI 3730 capillary platform (KAUST BCL) or a 3730*xl* DNA Analyzer (Applied Biosystems) at the Smithsonian’s National Museum of Natural History Laboratory of Analytical Biology. Sequences were deposited in the NCBI database with the accession numbers: MH338260-MH339541.

### Bioinformatics

#### Metabarcoding

We followed the bioinformatics protocol detailed by^62^. Briefly, raw reads from the sequencing machine were unzipped and denoised using the program BFC^63^. Contigs were formed between the forward and reverse reads using usearch (v8.1)^64^ with fastq_merge_maxee set to 1.0. Fastq files were converted to fasta files using mothur^65^. These were subsequently quality filtered and the forward and reverse primers removed using trim.seqs in mothur (maxambig = 0; maxhomop = 8; pdiffs = 0). Dereplication of the sequences was undertaken with the trie function in QIIME^66^. Sequences of the 18S gene were aligned in mothur while COI sequences were aligned using MACSE to remove reads with stop codons^58^. Reads were then pre-clustered (using the pre.cluster script with diffs = 3) and singletons, which are likely to be sequencing errors, were removed (split.abund with a cut off of 1). The remaining sequences were checked for chimeras (reference based using usearch) and clustered using CROP^67^ (COI: −l 3 and −u 4; 18S: using −s).

Reference sequences of the OTUs were taxonomically classified to the species level using Blast^68^ at the 97% similarity level against the BOLD^69^ and Midori^70^ databases for the COI gene and the PR2^71^ and Silva 128^72^ databases for 18S rDNA. Those reference sequences which did meet the designated cutoff were subsequently classified using the Statistical Assignment Program^73^ or rdp^74^ for the COI gene and 18S rDNA datasets, respectively.

#### Barcoding

Forward and reverse reads were combined into contigs using Geneious (Biomatters) with sequences trimmed if there was a greater than 5% chance of a base error. Sequences were also discarded if a stop codon was present in the translated sequence or the sequence had three ambiguous (N) bases resulting in the wrong amino acid translation. Sequences were clustered into OTUs using CROP (−l 3 and −u 4).

#### Diversity statistics

Alpha diversity statistics were calculated using the R (R development core team) package *phyloseq*^75^ and species accumulation curves were produced with the specaccum function in *vegan*^76^. Samples were sub-sampled to an even depth for comparisons (34,000 and 19,750 reads per sample for COI and 18S datasets, respectively). Alpha diversity statistics of the metabarcoding fraction were tested for significant differences (Kruskal-Wallis) in terms of two factors: shelf (three levels: near-, mid-shelf and off-shore) and fractions (three levels: sessile, 106–500 μm and 500–2000 μm). For the shelf factor analyses, only ARMS which contained all metabarcoding fractions were used (32 ARMS for COI and 26 ARMS for the 18S rDNA datasets. Differences in number of ARMS analyzed was due to some fractions not meeting thresholds for subsampling). Tests for multiple comparisons were undertaken with the Dunn test using the method proposed by^77^ implemented in the package *FSA*^78^. Due to the unbalanced nature of the sampling design, comparisons of unique OTUs were undertaken by selecting combinations of two reefs from each shelf position (all possible combinations were tested) and then calculating the average number of unique OTUs. The taxonomic composition of each sample was summarized in *phyloseq* with taxa merged at the phylum level and filtered so those groups representing on average less than 0.5% of the community were removed. Composition plots were produced using the package *ggplot*^79^. Similarly, PCoA plots were produced using *ggplot* with the ordination function of *phyloseq* on square root transformed data for both Jaccard and Bray Curtis dissimlarity matrices. Differences in the Jaccard and Bray Curtis values within and between reefs were tested using Kruskal Wallis. Using the PRIMER v6 package^80^ with the PERMANOVA + add-on^81^ a three-way PERMANOVA was undertaken for the metabarcoding fraction. This assessed the significance of the factors “Shelf Position” (orthogonal three levels, near-shore, mid-shelf and off-shore), “Fraction” (orthogonal three levels, Sessile, 106–500 μm and 500–2000 μm) and “Placement” (orthogonal two levels, Reef and Rubble). The statistical significance was tested using 9999 permutations of the residuals with a significance level of α = 0.05 under a reduced model. Significant effects were further investigated through a series of pairwise comparisons. The fractionated beta diversity, using the Jaccard measure, was calculated using the package *BAT*^82^ within the R framework. Total beta diversity (β_total_) was partitioned into its two components, richness (β_rich_) and replacement (β_repl_), as described by^83^. The decomposition of the Jaccard dissimilarity was performed for the combined ARMS (all metabarcoded fractions combined) and each fauna component separately (Sessile, 106–500 μm, 500–2000 μm and >2000 μm). Triangle plots were used to provide a two-dimensional illustration of the relationships among the two components of beta-diversity in analysis (replacement, richness difference) and the Jaccard similarity index^84^. Each vertex of the equilateral triangle corresponds to one of the coefficients and the sum of them equals 1. Each dot represents a pair of samples and its position is proportional to the respective coefficient values^84^. The density of the dots illustrates the structure of the data in terms of faunal similarity, replacement and richness difference. The higher the values associated with each of the vertices, the more relevant the corresponding coefficient is. Plots were created for: (1) between all samples (all pairwise comparisons possible); (2) between samples belonging to the same sites (within-site variation); (3) between samples belonging to different sites (among-site variation); (4) between samples belonging to the same shelf position (within-shelf variation); and (5) between samples belonging to different shelf positions (among-shelf variation). Local Contributions to Beta Diversity (LCBD) as described by^85^, i.e., the degree of uniqueness of the sites in terms of species richness, was calculated in the *adespatial* package in R^86^. Total diversity was calculated on a Hellinger transformed community matrix. Beta diversity was partitioned computing the sums of squares of a sampling unit as a proportion of the total diversity^85^. Linear distances were calculated between pairs of reefs and plotted against similarity (defined as 1 – Jaccard/Bray Curtis dissimilarity).

To assess the correlation in dissimilarity patterns between the different components of the fauna (sessile and mobile fractions of the metabarcoding), comparative (Mantel-type) tests were produced based on the Jaccard dissimilarity matrices using the *vegan* package in R. Similar tests were undertaken to assess the patterns produced in the metabarcoded datasets using the two different primer sets. To assess the differences in the number of OTUs per taxon both primers sets were subsampled to 19750 reads for the purpose of the comparison.

### Data accessibility

Sequences from the barcoding are deposited in the NCBI database under the following accession: MH338260-MH339541. The raw sequences from the metabarcoding are deposited in Figshare at the following: 10.6084/m9.figshare.5559106.v1, 10.6084/m9.figshare.5558113, 10.6084/m9.figshare.5549365, 10.6084/m9.figshare.5552782, 10.6084/m9.figshare.5552932.

## Results

### Environmental variability

SST varied between 28 °C (January and March) and 34 °C (August) with no differences observed among reefs (Supplementary Figure S1 and Supplementary Table S3).

Chlorophyll *a* data showed a more variable pattern based on the reef category. In general, the lowest values were observed for the two off-shore reefs (Abu Madafi and Shib Nazar). The near-shore reef East Fsar had the highest values throughout the two-year period. The two other near-shore reefs, Abu Shosha and KAEC, had low values of chlorophyll *a* (the data for KAEC was, however, sparse), while the mid-shelf reefs showed intermediate concentrations of chlorophyll *a* (Fig. 1 and Supplementary Table S3).

### Biodiversity patterns

#### Alpha-diversity

After sub-sampling at an even depth (34,000 and 19,750 reads per sample for COI and 18S datasets, respectively), a total of 5420 OTUs and 3830 OTUs were obtained with the COI and 18S primers, respectively. Metabarcoding of the combined biological fractions of ARMS showed an average of 660.46 ± 151.50 OTUs for COI and 750.03 ± 106.97 OTUs for 18S (Supplementary Table S4).

**Figure 3:**
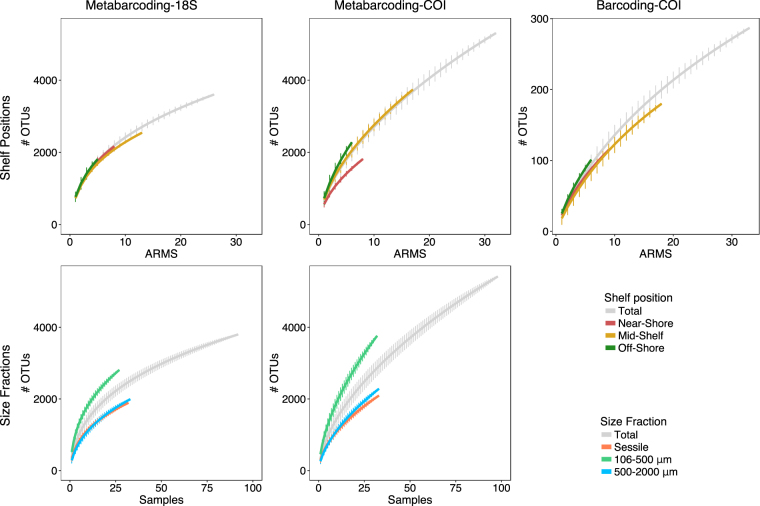
Species accumulation curves computed per size fractions (Sessile, 106–500 μm, 500 μm) and shelf positions (Near-shore, Mid-shore, Off-shore). Note that we used DNA metabarcoding to characterize communities of organisms <2000 um and DNA barcoding for communities >2000 um.

Combining the whole dataset, a total of 1023 OTUs (26.7%) were shared amongst the different size fractions in the 18S dataset, whereas 872 OTUs (16.0%) were shared in the COI dataset. Statistically significant differences were observed in alpha-diversity among the size fractions (chi-squared = 45.358, P < 0.001 for 18S; chi-squared = 44.978, p < 0.001 for COI). For both primer sets, the 106–500 μm size fraction was the most diverse (18S: 522.18 ± 127.82; COI: 456.46 ± 113.03) and had the highest number of unique OTUs (Table 1). This fraction also made the largest contribution to total biodiversity (Fig. 3). Sessile and 500–2000 μm size fractions contributed to a lesser extent and similarly to the total biodiversity (Fig. 3). The diversity was under-sampled, as the rarefaction curves did not reach a plateau (Fig. 3).

**Table 1:** Cross-shelf differences in OTU diversity for both the 18S and COI datasets.

18S	Near-shore					Mid-shore					Off-shore				
	Metabarcoding				Barcoding	Metabarcoding				Barcoding	Metabarcoding				Barcoding
	Sessile	106–500	500–2000	Total	>2000	Sessile	106–500	500–2000	Total	>2000	Sessile	106–500	500–2000	Total	>2000
# OTUs	1144	1592	1056	2227	NA	1417	1545	1494	2578	NA	830	1512	793	1915	NA
Mean #OTUs	335	542	309	749	NA	316	469	276	708	NA	325	527	275	748	NA
Mean rarefied # OTUs	335	542	309	772	NA	331	503	274	730	NA	325	544	275	768	NA
Unique OTUs	118	268	119	551	NA	208	361	209	902	NA	60	236	77	374	NA
**COI**	**Near-shore**					**Mid-shore**					**Off-shore**				
	**Metabarcoding**				**Barcoding**	**Metabarcoding**				**Barcoding**	**Metabarcoding**				**Barcoding**
	**Sessile**	**106–500**	**500–2000**	**Total**	**>2000**	**Sessile**	**106–500**	**500–2000**	**Total**	**>2000**	**Sessile**	**106–500**	**500–2000**	**Total**	**>2000**
# OTUs	702	1401	879	1900	120	1632	2580	1638	3739	188	851	1659	818	2278	104
Mean # OTUs	221	402	250	556	23	330	466	263	691	20	306	510	260	730	25
Mean Rarefied # OTUs	221	402	250	556	NA	330	466	263	691	NA	306	510	260	730	NA
Unique OTUs (Rarefied)	89	321	136	586	53	457	958	441	2142	104	150	516	157	882	55

Mean number of OTUs is per ARMS. Note that the largest size fraction was only analyzed with COI.

**Figure 4:**
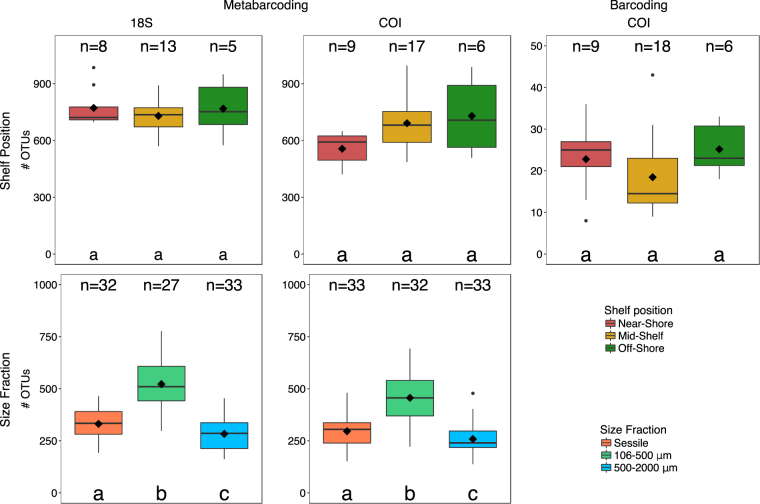
Differences in average number of OTUs per ARMS among size fractions (Sessile, 106–500 μm, 500–2000 μm) and shelf positions (Near-shore, Mid-shore, Off-shore). The average for the shelf positions was taken from those ARMS that had all size fractions present. The median is shown by the solid line across the box whilst the mean is indicated by the black diamond. Small dots represent outliers. Letters denote those categories, which are based on the DunnTest. n is the number of samples per shelf or size fraction.

In terms of shelf position, little variability in alpha-diversity (i.e. number of OTUs) was detected for either the 18S or COI data (chi-squared: 0.735 and 5.776; p = 0.69 and 0.55 for the 18S and COI, respectively) (Fig. 4 and Table 1). Off-shore reefs had the most distinctive OTUs (472 and 530 unique OTUs for the COI and 18S datasets, respectively) for both primer sets. However, as a proportion of observed OTUs, unique OTUs accounted for a higher percentage in the near-shore (22.3% compared with 20.7% Off-Shore) for the COI dataset. For the 18S and the COI primers, respectively, 28.7% and 15.0% of the OTUs were shared amongst all shelf positions. The near-shore and off-shore reefs shared fewer OTUs than the near-shore and mid-shelf reefs. There was no significant correlation between the number of OTUs and chlorophyll *a* values (F = 0.4255, p = 0.5197). In terms of differences in OTUs between the two placements, pairwise comparisons revealed no significant difference in OTU number within a reef position.

In total, there were 287 OTUs for the >2000 μm (barcoded) fraction. Only 22 (7.7%) of these were shared among all areas, with the mid-shelf reefs having the highest proportion of exclusive OTUs (34.4%). The near- and off-shore reefs shared the fewest OTUs. On average, there were 20.85 ± 8.64 barcoded OTUs per ARMS and no clear pattern across the shelf gradient was detected (Fig. 4).

#### Community composition and structure

**Figure 5:**
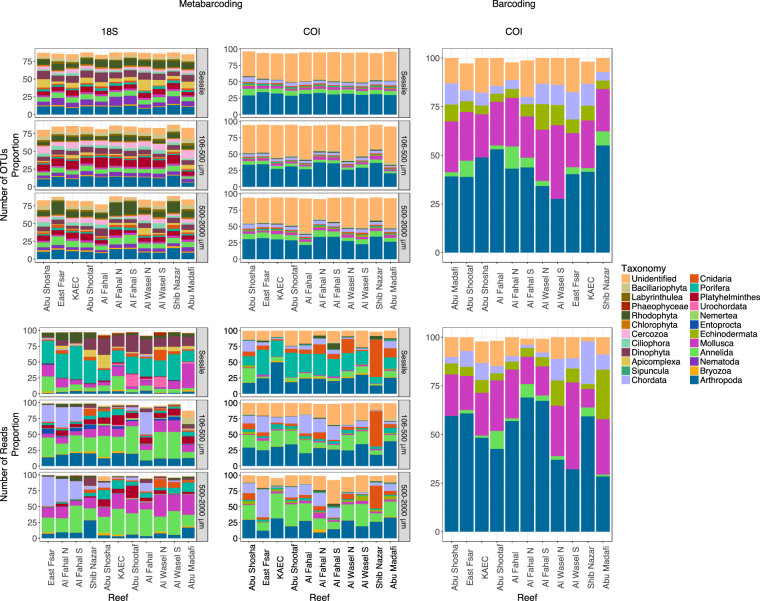
The structure (# of OTUs; top row) and composition (abundance of reads; bottom row) of communities at each reef. Taxa were filtered at 1% for the structure and 0.5% for the composition, which means that proportions do not sum to 100%.

In the COI dataset, a high proportion (~42%) of the OTUs could not be assigned to any taxonomic group; however, these were generally low abundance OTUs, as only 18.4% of reads were unassigned. Arthropoda contributed the highest number of assigned OTUs in all fractions. However, Porifera accounted for a higher proportion of reads (25.8%) than Arthropoda in the sessile fraction. In the mobile fraction, Annelida had a small number of assigned OTUs although they accounted for a high proportion of reads (20.0%). Similarly, the species richness (number of OTUs) of Chordata was low but in various reefs (especially East Fsar and Al Fahal) they made substantial contributions to the total number of reads (Fig. 5 and Supplementary Figure S2).

In the 18S dataset, Arthropoda were not as dominant in terms of either OTUs or reads, compared to the COI dataset. Arthropoda were almost absent from the sessile fraction (3.0% in terms of reads) and only had an average of 15.6% of the reads (compared to 28.2% in the COI) in the 106–500 μm fraction. In the sessile fraction, Porifera was the dominant group (29.7% of reads), and Dinophyta accounted for ~15% of reads. Annelida accounted for a high proportion of reads (28.7%) of the mobile fractions, as did Mollusca (11.7% and 20.2% for the 106–500 μm and 500–2000 μm size fractions, respectively). Some groups, such as Cercozoa and Rhodophyta, had relatively high numbers of OTUs but these did not account for a high proportion of reads (Figs 5 and S2).

In general the ARMS placed on the reef framework had a higher proportion of Chordata except in Shib Nazar, which was dominated by Cnidaria. Al Fahal, which had ARMS placed in both microhabitats (reef and rubble), interestingly had higher proportions of Chordata in both sets of ARMS. In contrast the ARMS positioned in rubble had a higher proportion of Annelida especially in the COI dataset. In the sessile fraction of the 18S rDNA dataset Dinophyta were more prevalent in the rubble ARMS.

In the barcoding of the >2000 μm fraction, members of the Arthropoda were the dominant taxa at all reefs with the exception of Shib Nazar (for both OTU counts and abundance) (Fig. 5). Other taxa contributing substantially to the composition of the >2000 μm fraction included Mollusca across all stations, and Chordata especially in Al Wasel North and at the two offshore reefs (Shib Nazar and Abu Madafi).

As expected based on primer versatility, differences were noticed in the taxonomic composition of the community using the two target genes (Supplementary Table S5). For instance, the 18S rRNA gene dataset revealed Dinophyta to be a substantial contributor to the sessile fraction. However, it was absent from the COI data, which reflects the broader taxonomic range of the ribosomal unit. There were also differences in the proportion of metazoan reads recovered. Echinodermata and Sipuncula were observed in the COI dataset at average proportions greater than 0.5% but were negligible in the 18S rRNA gene dataset (on average <0.25%). On the other hand, Platyhelminthes accounted for a minor component in the mobile fractions in the 18S dataset, but were absent from the COI data. Despite these differences in detection efficiency, Bray Curtis and Jaccard distance matrices built with the markers were not significantly different based on Mantel tests (r = 0.412; p < 0.001 for Bray-Curtis and r = 0.578; p < 0.001 for Jaccard).

#### Beta-diversity

**Figure 6:**
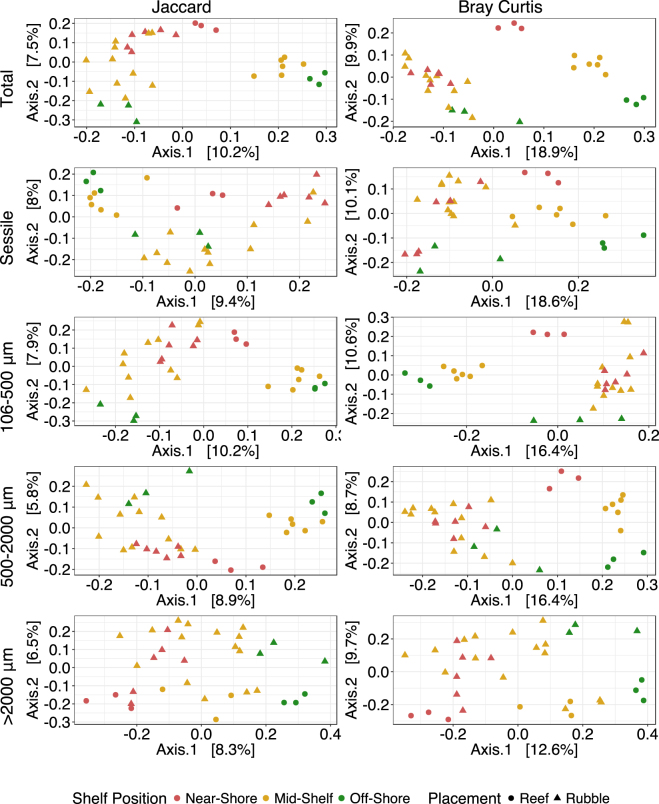
PCoA analysis illustrating dissimilarities in community composition based on Jaccard and Bray Curtis dissimilarity matrices of COI. Analysis was undertaken on the full ARMS unit as well as the different fractions (Sessile, 106–500 μm and 500–2000 μm and >2000 μm). Points are coloured according to shelf position.

Within and between reef comparisons indicated that within reef similarity was higher than between reef similarity (p < 0.001). PCoA analysis indicated that both the structure and composition of the ARMS communities differed across the shelf (Figs 6 and S3). For both the Bray Curtis and Jaccard analysis significant interactions were observed between the factors, suggesting inconsistent variation in the trends (Supplementary Table S6). Pairwise comparisons showed that in general the community differed significantly in terms of shelf position and fraction (Supplementary Table S6). As patterns are similar, the COI data are shown with the 18S rRNA gene data being provided in supplementary data (Figs 6 and S3 and Supplementary Table S6). Further, there was a split between the different microhabitat placements. This pattern was observed regardless of the primer set or the molecular approach used (i.e. metabarcoding or barcoding).

**Figure 7:**
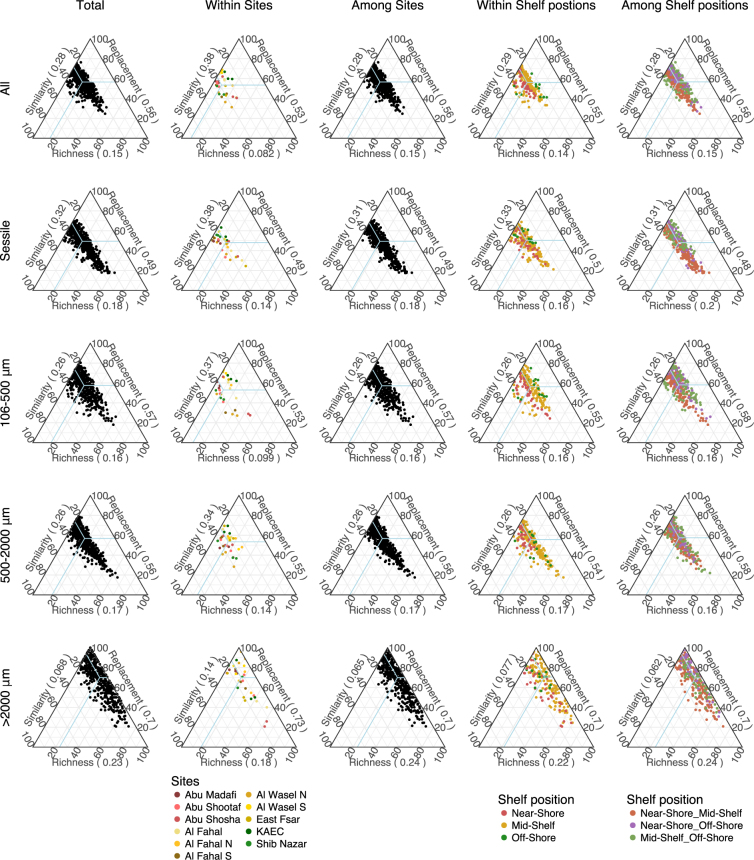
Ternary plots of similarity [1-D (Dissimilarity obtained using Jaccard)] and the partitions of beta diversity (replacement and richness) for the full ARMS community and the various fractions obtained from the COI metabarcoding and barcoding. Ternary plots are shown for the total experiment as well as within and among sites and within and among shelf positions. Numbers in brackets on the axis labels represent the mean value.

Considering all the fractions and units, and for both primers, pairwise comparisons between samples indicated that between 28 and 46% of species were shared (Figs 7 and S4: Total: All). Higher variability was observed for the 106–500 μm size fraction, whereas the barcoded fraction showed the lowest similarity in species composition (*i.e*., the highest beta-diversity) (Figs 7 and S4). However, the mean values of similarity were rather consistent for the metabarcoding fractions. Slightly lower similarity was detected in the mobile assemblages compared to the sessile assemblages. In addition, similarity values dropped with the increase in spatial scale (from within-reef comparisons to cross-shelf comparisons) (Figs 7 and S4). Nevertheless, mean similarity values within sites for each one of the assemblages assessed using metabarcoding techniques (sessile: 0.38; 106–500 μm: 0.37; 500–2000 μm: 0.33) indicate considerable changes in the species composition at the smaller spatial scale (*i.e*., the reef). For the largest mobile size fraction (>2000 μm), the maximum average similarity detected at the scale of the reef was 0.22 (*i.e*., 78% dissimilarity), indicating high heterogeneity in the species composition at the finer spatial resolution (<10 m).

Partitioning of the beta-diversity into its replacement and richness components showed that most of the beta-diversity was accounted for by replacement with only a minor contribution from richness. When considering all the possible combinations (i.e. Total:Metabarcoding All), mean values of pairwise similarity indicated that 72% of the species were observed in a single ARMS unit. On average the majority (56%) of the beta-diversity was due to changes in the identity of the community (replacement). Only 15% of OTUs were unique to the richest ARMS sample. The contribution of richness is slightly higher for the >2000 assemblage yet still low, ranging from 18% for within-sites comparison to 24% between-shelf position (Figs 7 and S4).

In the near-shore sites, the ratio replacement:richness was highest and decreased toward the off-shore, indicating richness had more of an impact on beta diversity in the offshore stations than the near or mid-shelf reefs (Supplementary Table S7).

Mantel tests on the correlation between the sessile fraction and the various size fractions of the motile fauna showed significant positive correlations of all possible combinations (p < 0.001). However, there was a decline in R value with increasing size fraction, from 0.5886 (106–500 μm) to 0.5265 (500–2000 μm) to 0.4251 (>2000 μm).

**Figure 8:**
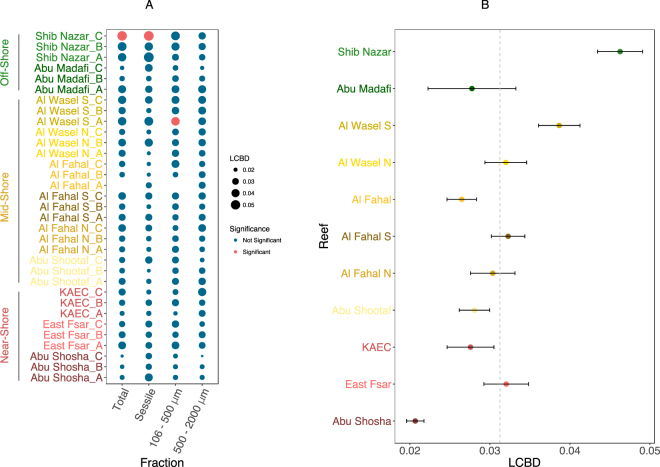
Local Contribution to Beta Diversity (LCBD) values. (**A**) The individual replicates. Values are depicted for the COI metabarcoding of the full ARMS and the various fractions. The size of the circle is proportional to the LCBD value and red indicates a significant difference. (**B**) The average LCBD for the full ARMS per reef. The dotted line in (**B**) indicates the value of LCBD if all reefs contributed equally.

The contribution of each reef to the overall beta-diversity of reef cryptic benthic assemblages, assessed using LCBD indices, indicated that only a few replicates had a significant contribution to the overall beta-diversity (Fig. 8A). Reefs did not contribute evenly and no clear patterns were detected according to their position on the shelf. LCBD ranged from 0.021 for the near-shore reef Abu Shosha to 0.046 for the offshore reef Shib Nazar (Fig. 8B). Five out of 11 reefs sites showed contributions above the average.

There was a negative relationship between the linear distance between reefs and similarities in community composition (Supplementary Table S8) regardless of primer, dissimilarity equation or size fraction analyzed. However, variability amongst the data was high with low R^2^ values being observed.

## Discussion

The use of ARMS combined with molecular methods on a cross-shelf gradient in the central Red Sea has revealed important insight into the hidden majority of biodiversity in coral reef habitats. We identified thousands of OTUs across our sites. Most sites had sessile communities dominated by representatives of Porifera while Arthropoda and Annelida were important components of the mobile fraction. Distinct cross-shelf gradients were observed for both species composition and structure, with replacement contributing the most to beta-diversity. Similar trends in community patterns were observed for both the COI and 18S genes.

Traditional transect surveys undertaken in the Red Sea reveal between 1–100 coral species per site^87–89^ with 54–70 fish species being present^88^. In the current study the extent of the hidden majority of cryptic fauna was observed with on average, 660 and 750 OTUs per ARMS and a total of 5420 OTUs and 3830 OTUs for the COI and 18S metabarcoding datasets, respectively. Of the totals, 2042 and 1245 OTUs, respectively, were invertebrates giving a greater or similar number of OTUs to a recent attempt to create an species inventory (albeit incomplete) of non-coral invertebrates in the whole of Red Sea^90^. While it is acknowledged that metabarcoding studies can over estimate the diversity present in a ecosystem^91,92^, the high alpha and gamma (i.e. total number of OTUs) diversity observed in this study within a single region confirms: (i) the overwhelming diversity of cryptic species present in the reef that has been overlooked^44,45^, and (ii) the importance of supplementing traditional survey methods (i.e. visual surveys) with DNA-based surveys in order to broaden the taxonomic scope of reef monitoring programs. ARMS sampling allows for the quantification of the diversity of organisms that depend on the three-dimensional structure present in reef habitats, especially species living hidden among and on coral rubble.

Porifera was the most prominent sessile component on the ARMS in agreement with other ARMS in the central Red Sea^45^, but in contrast to that reported for the Gulf of Aqaba where algal groups had higher contributions^44^. The presence of Porifera on the ARMS is expected due to their high abundance and diversity in cryptic spaces of the reef^38,39^. These sponges most likely represent an important habitat for vagile species recorded in this study. The use of the 18S rRNA gene allowed for the identification of dinoflagellates, primarily the symbiotic genus *Symbiodinium*, to be a substantial component of the sessile fraction. In agreement with the other ARMS studies in the Red Sea, Annelida and Arthropoda played major roles in the composition and structure of the mobile community^45^.

While the overall patterns were similar between faunal components, there was an inverse relationship between organism size and community similarity, with the largest and most vagile assemblages being the most dissimilar at the scale of our study. Furthermore, the partitioning of beta diversity indicates that replacement increased with size. Indeed, Mantel test comparisons between the sessile component and the various size fractions indicated a decreasing relationship with an increase in the size fraction. This suggests that the structuring factors appear to be different for the largest organisms assessed. While this may be related to the different methodological approach used for this fraction (*i.e*., barcoding versus metabarcoding, which samples organisms in the gut or growing on surfaces as well) it is likely that the main differences are related to responses to different biological (such as mobility and dispersal behavior, predation) or environmental parameters^93,94^.

### Cross-shelf patterns

Benthic communities are structured at multiple scales depending on global-, regional- (currents, affecting dispersal patterns) and local- (such as biological interactions, niche availability) scale processes^93,95^. Dispersal patterns of organisms within a reef system are affected by the larval behavior of the dispersing organisms and the prevailing currents in the region^96^. A significant negative relationship between linear distance and similarity was shown in the current study, although a low R^2^ value suggests that distance *per se* is unlikely to play a driving role in community patterns at the scales studied. This would be in agreement with previous studies, which showed that changes in sponge communities were not accounted for by distance^97,98^. However, linear distance does not necessarily accurately reflect complexity of connectivity potential between sites^99^. Prevailing currents often have complex patterns and specific formations, such as boundary currents, may affect dispersal across a shelf^100^. Cross-shelf patterns have previously been reported for large sessile organisms within coral reefs (e.g.)^6,14,21,101,102^ and algal biofilms^9^. In our study region, cross-shelf shifts have been documented in fishes^88^, in scleractinians^10^ and algal biofilms^9^. However, the responses of cryptic fauna to this gradient has previously not received much attention.

Regardless of the primer used or the size fraction examined, differences in both the composition and structure of the ARMS community were documented across the shelf, as hypothesized. Previous studies in other regions of the world have suggested that environmental factors such as water quality^6,103,104^, sedimentation^101,105,106^ and/or nutrient enrichment^107–109^ influence the ecology of the benthic assemblages of coral reefs. Indeed elevated near-shore nutrient concentrations have been found to be a factor in structuring benthic communities in the southern Red Sea^8^. While assessments of these factors were out of the scope of the current study, satellite data indicated that off-shore reefs were associated with the lowest levels of chlorophyll *a*. Values at the near-shore reefs were variable (high for East Fsar but low for Abu Shoosha) with mid-shelf reefs having values intermediate between East Fsar and the off-shore reefs. Differences in chlorophyll *a* concentrations are likely to indicate differences in nutrient availability on the reefs, as well as variations in the concentrations of organic matter within the water column. Alterations in the concentrations of organic matter are likely to have an effect on various benthic organisms such as those that are predominantly filter feeders or detritivores. The collection of *in-situ* measurements for a variety of environmental variables during the period of the submersion of the ARMS units at each reef would allow for a better characterization of the environmental factors affecting the reef community.

To date, only a few studies have explicitly measured the partitioning of beta-diversity in reef environments^110–112^. Overall, in the present study, it was observed that the contribution of sites to the overall diversity generally increased towards the off-shore with Abu Madafi, in particular, having a unique species composition. This is further exemplified by the fact that the off-shore position had on average the highest number of unique OTUs, although in the COI dataset this could partially be explained by the higher number of OTUs observed.

Compartmentalizing the beta diversity suggested, regardless of the primer used, that species replacement was the predominant component of beta diversity and richness differences only made a limited contribution. The contribution of richness to the overall biodiversity was highest within the off-shore sites, maybe resulting from the higher number of observed OTUs in Abu Madafi compared to Shib Nazar. The high contribution of replacement (i.e. substitution of species) suggests that the structural complexity of the reefs produces a wide variety of microhabitats that can be colonized by a myriad of organisms, which has been observed for other habitats in the Red Sea^113^. Also, it could indicate that dispersal of the organisms between reef sites is limited by processes such as coastal boundary layers^100^, as it is proposed that high connectivity results in homogenization of the community^114^. The variability in niches allows for the potential co-existence of species with the same ecological roles/functions, which would promote the resilience of the reefs (high levels of redundancy). This is exemplified by the high variability in beta-diversity observed between reefs sharing the same position on the shelf as well as within sites. A range of factors including light, sediment, wave energy, substrate stability and biological interactions^115^ influence the diverse nature of the cryptic environment. Microzonation of cavity dwellers (coelobites) has been previously shown in coral rubble^116^ and across a single reef^117^. High variation in species composition of coral-dwelling invertebrates has been shown among different coral families^118^. This suggests that there is a degree of host specificity within the coral invertebrate community^118^, which may reduce competition and allow for greater resource partitioning^119^, which promotes replacement.

### Differences between ARMS sets

Although there were clear cross-shelf effects in the dataset, there was also a difference in the composition and structure between the two microhabitats where ARMS were placed. This difference was observed for all fractions of the dataset. While this could have just been due to the broader spatial scale of assessed reefs for those ARMS placed on rubble, the fact that the two microhabitats on Al Fahal S differed suggests other factors affected the community composition. While the current study is unable to determine the causes of these differences there are two possibilities: small discrepancies in the microenvironment of the deployment habitat between the two ARMS sets as well as differences in the timing of deployment and retrieval of the two sets. Although all ARMS were positioned on the exposed side of the reef, other aspects of positioning within the reef structure were different, particularly whether the ARMS were positioned on the coral framework or on coral rubble. Thus, factors such as the input of sediment or the local community pool, for instance, could have affected the composition of the benthic community. Also it cannot be ruled out that temporal factors could impact the community present on the ARMS. The use of molecular methods allows for the detection of eggs and larvae, which may not be visible in morphological studies. While little is known about the spawning periods of benthic fauna, with the exception of some coral and echinoderms^120,121^, colonization of ARMS plates by eggs or larvae and the predators consuming them could have an impact on the community assemblages.

### Primer comparisons

There is a well-recognized trade-off between taxonomic resolution and amplification efficiency among metabarcoding markers. Recently, Leray and Knowlton^57^ recommended targeting the slowly evolving 18S rRNA gene region to get a broad overview of the eukaryotic domain followed by the amplification of a lineage-specific hypervariable marker such as COI for higher taxonomic resolution among metazoans. Previous studies characterizing marine diversity from ARMS or other habitats have typically targeted either the 18S rRNA gene^45^ or the COI gene^42^, but never both. Here, as expected, we showed differences between the two primer sets in the taxa, which we could annotate taxonomically. This could be either due to differences in amplification efficiency or reference sequences being missing from the appropriate databases and thus amplified sequences not being assigned the correct taxonomic group^42^. For example, in the current dataset, Alveolata were not observed at all in the COI dataset despite accounting for large numbers of OTUs in the 18S rRNA gene data. On the other hand there was a substantial increase in the number of observed Arthropoda OTUs in the COI dataset compared to the 18S rDNA likely linked to the bias in the COI reference libraries towards this taxon. Despite these differences in both the taxonomy and the observed number of OTUs, comparative Mantel tests (Spearman’s rank) showed that the diversity patterns obtained from both primer sets were not significantly different. This suggests that whilst using two primer sets might be useful for obtaining a better understanding of the taxa present in the community in agreement with previous studies^59,122,123^, the use of a single primer may be adequate for detecting biodiversity patterns as these broad scale patterns appear to be independent of marker choice^122,124^.

## Conclusion

We observed that across the shelf and biological components, the underlying processes shaping cryptic biodiversity are consistent and driven by spatial turnover (or replacement). The high replacement observed within reefs may suggest low levels of connectivity and a high heterogeneity in niches present at the scale of the reef. This combined with the possibility of a high degree of host specialization among coral-dwelling invertebrates^118^ promotes the coexistence of a myriad of species and the high rates of replacement observed.

Conservation efforts are often limited by funding and appropriate selection of protected areas is vital. In this case, patterns of beta-diversity can have an impact on the debate. The current study revealed the prominence of replacement processes in the partitioning of beta-diversity for the cryptic organisms. Thus, to best conserve the diversity within the reefs and to minimize species loss, a network of reefs must be protected rather than just the most species-rich reef ^ 2,125^. The present study reinforces the importance of taking into account beta-diversity patterns to better understand the ecological changes across environmental gradients in reef systems, as had been suggested before for other habitats^2,26,126^ and for many of our study reefs^88^. The incorporation of beta-diversity analysis at multiple spatial scales in ecosystem-based management approaches is recommended in order to attain the long-term conservation of the reef biodiversity and the overall ecosystem health^115^.

Finally, we showed that the two primer sets amplified different fractions of the cryptobenthic community, but the overall diversity patterns were similar. Currently, one of the major limiting factors in metabarcoding studies is the incomplete nature of reference databases, and the future development of these databases will likely dictate the accuracy of taxonomic assignments and improve ecological interpretations.

With marine habitats under severe pressure it is vitally important for conservation that changes in biodiversity patterns can be detected using standardized methodologies. In this study we demonstrated the ability to detect changes in the composition and structure of the cryptic fauna, a vital component of reef diversity across a cross-shelf gradient.

## Electronic supplementary material

Supplementary Information
