# Supplementary material for: Cross-shelf investigation of coral reef cryptic benthic organisms reveals diversity patterns of the hidden majority
Source: Sci Rep. 2018 May 24;8:8090. doi: 10.1038/s41598-018-26332-5 (PMC5967342; doi:10.1038/s41598-018-26332-5)
Supplement: Supplementary file 1 — Supplementary Information [file 41598_2018_26332_MOESM1_ESM.pdf]

Cross-shelf investigation of coral reef cryptic benthic organisms reveals diversity patterns of the hidden majority

Pearman, J.K.[1*], Leray M[2], Villalobos R[1], Machida R.J[3], Berumen M.L[1], Knowlton, N[4], Carvalho, S[1].

[1] King Abdullah University of Science and Technology (KAUST), Red Sea Research Center (RSRC), Biological and Environmental Sciences and Engineering (BESE), Thuwal, 23955-6900, Saudi Arabia
[2] Smithsonian Tropical Research Institute, Panama City, Balboa, Ancon, Republic of Panama
[3] Biodiversity Research Center, Academia Sinica, Taipei, Taiwan
[4] National Museum of Natural History, Smithsonian Institution, Washington, DC USA

*corresponding author email: john.pearman@kaust.edu.sa

Supplementary Figure S1: *Monthly variations in sea surface temperature derived from NASA's Oceancolor website (https://oceancolor.gsfc.nasa.gov/) derived from the MODIS A satellites at a 4km resolution. Al Fahal S and Al Wasel sites were combined as they were not greater than 4km apart and data for KAEC was unavailable.*

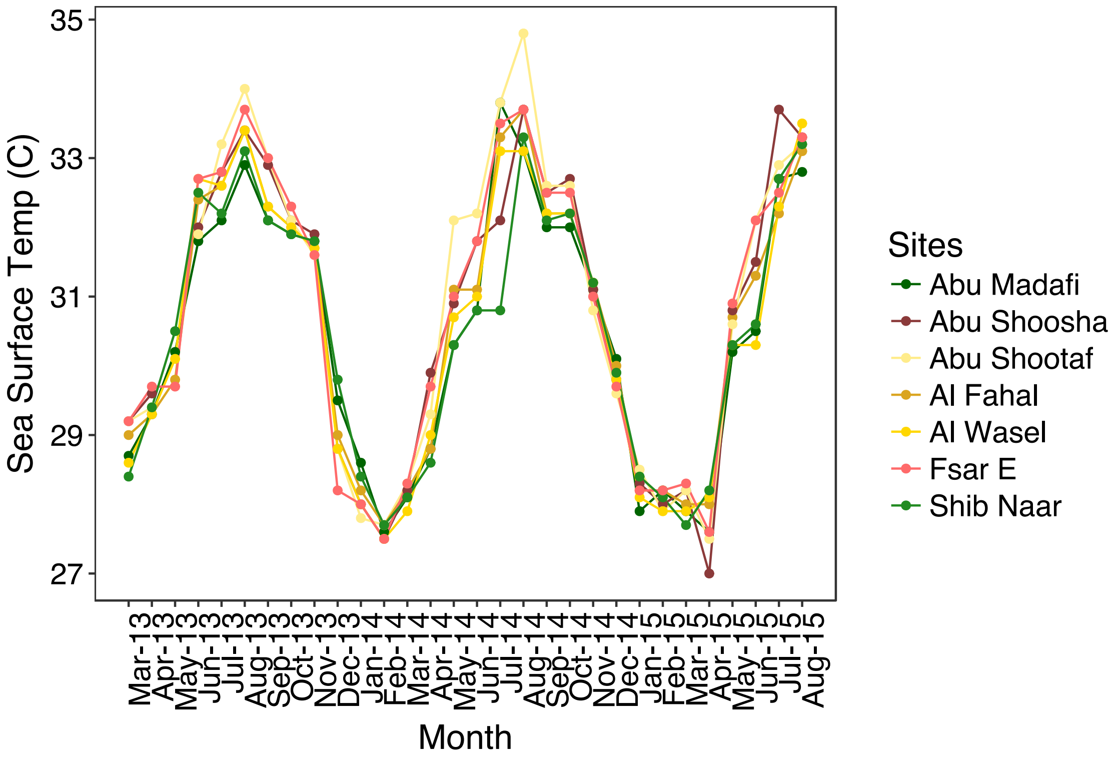

Figure S2: Composition (abundance of reads) of metazoan communities at each reef. Taxa were filtered at 0.5%, which means that proportions do not sum to 100%.

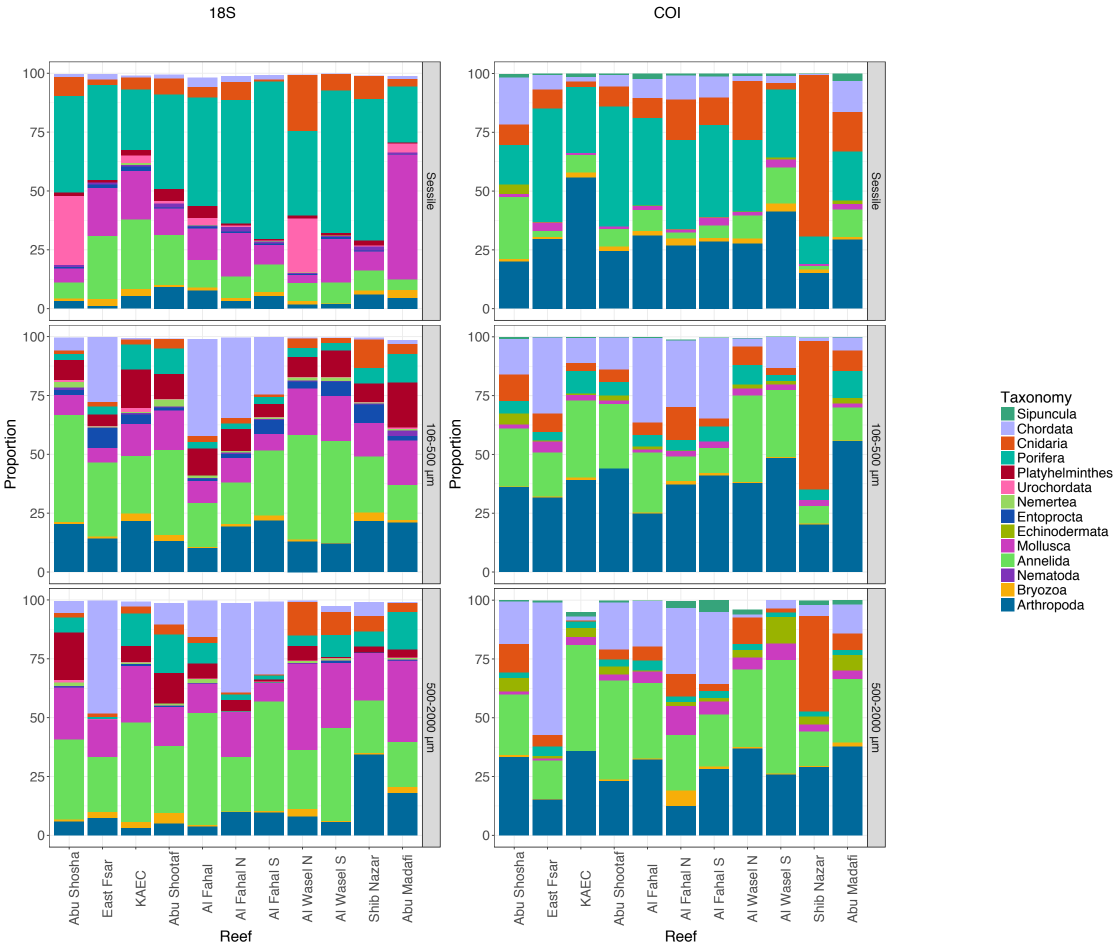

Figure S3: PCoA analysis of the composition of the reef communities based on Jaccard and Bray Curtis dissimilarity matrices from the 18S metabarcoding dataset. Analysis was undertaken on the full ARMS unit as well as the different fractions (Sessile, 106-500 µm and 500-2000 µm). Points were colored according to shelf position.

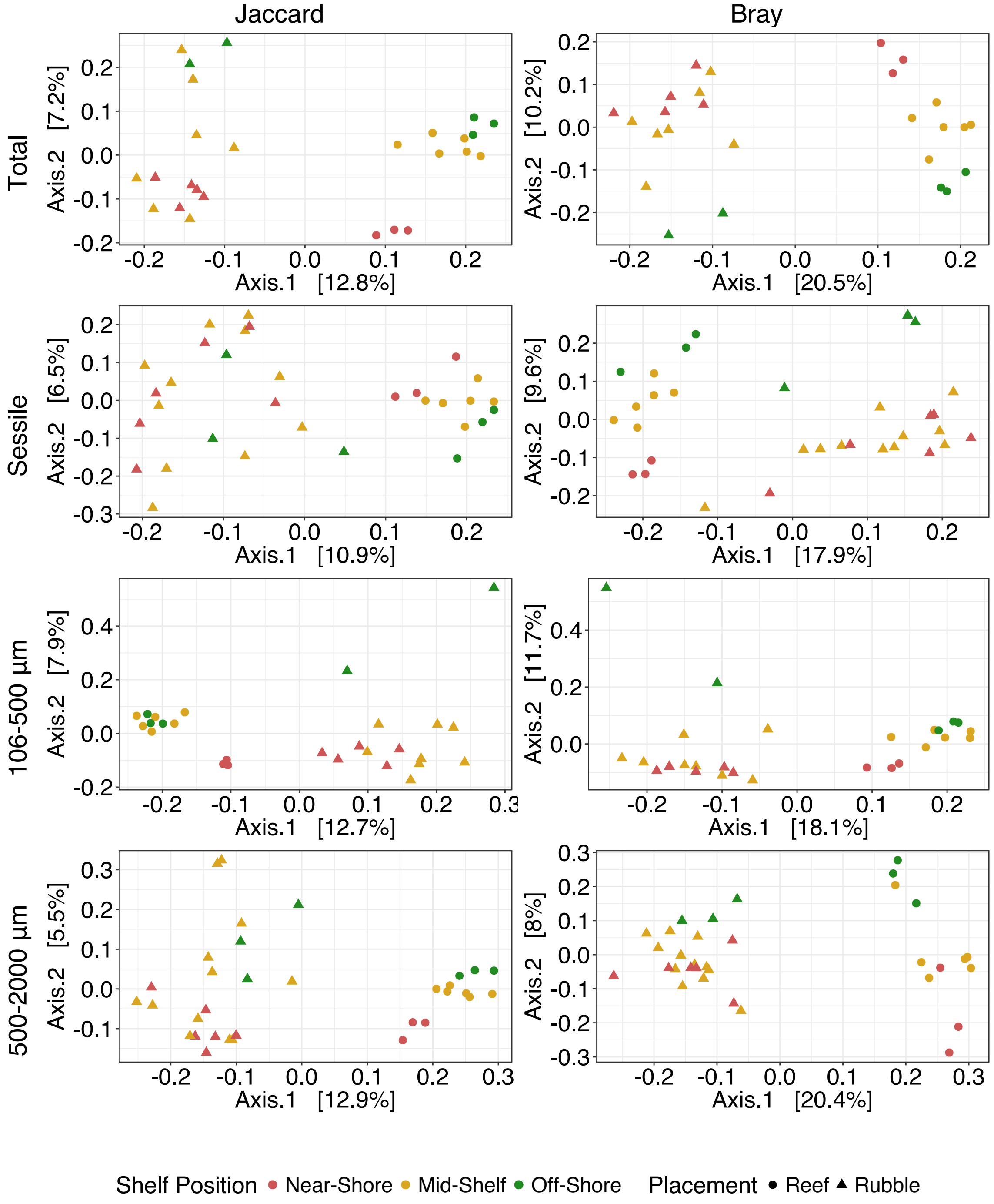

Figure S4: Ternary plots of similarity [1-D (Dissimilarity obtained using Jaccard)] and the partitions of beta diversity (replacement and richness) for the full ARMS community and the various fractions obtained from the 18S metabarcoding. Ternary plots are shown for the total experiment as well as within and among sites and within and among shelf positions. Numbers in brackets on the axis labels represent the mean value.

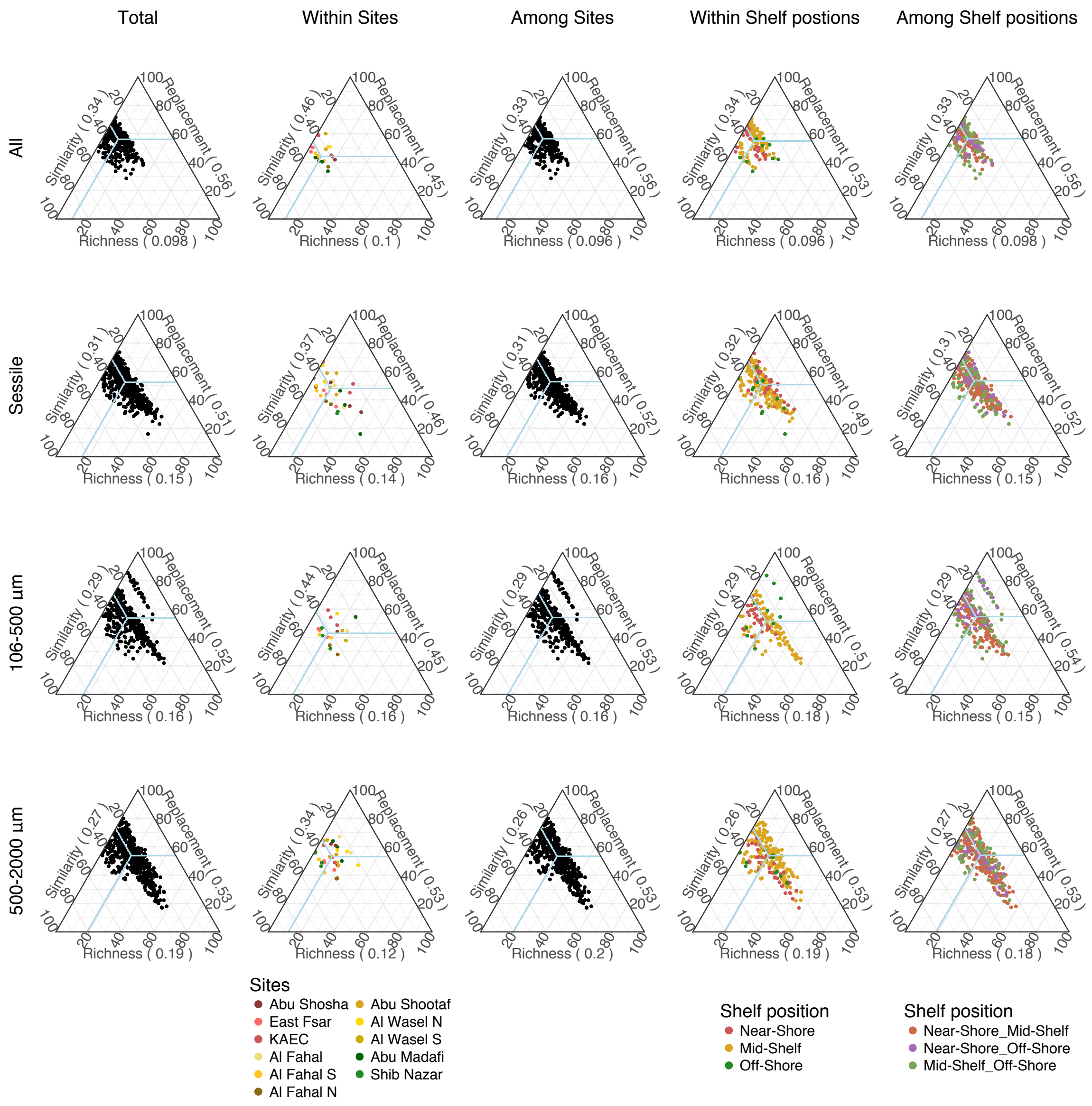

Supplementary Table S1: Longitude and latitude coordinates for each of the sampled reef sites

	Longitude	Latitude	Depth (m)	Deployed	Retrieved	Placement
Abu Shosha	39.0442	22.2998	12	May 2013	May 2015	Coral rubble
East Fsar	39.0286	22.2307	10	Febuary 2013	May 2015	Reef framework
KAEC	39.0602	22.3698	12	June 2013	July 2015	Coral rubble
Abu Shootaf	38.9676	22.1387	9	June 2013	May 2015	Coral rubble
Al Fahal N	38.9601	22.3015	10	Febuary 2013	May 2015	Reef framework
Al Fahal S	38.9650	22.2238	10	Febuary 2013	May 2015	Reef framework
Al Fahal	38.9650	22.2238	10	May 2013	May 2015	Coral rubble
Al Wasel N	38.9400	22.6792	12	June 2013	June 2015	Coral rubble
Al Wasel S	38.9585	22.6616	10	June 2013	June 2015	Coral rubble
Abu Madafi	38.7781	22.0893	10	May 2013	May 2015	Coral rubble
Shib Nazar	38.8550	22.3205	10	Febuary 2013	May 2015	Reef framework

Supplementary Table S2: Nucleotide sequences of the primers used in this study for the COI and 18S rRNA metabarcoding and the COI barcoding

Metabarcoding		Forward	Reverse
	COI	GWACWGGWTGAACWGTWTAYCCYCC	GTAIACYTCIGGRTGICCRAARAAYCA
	18S	CCAGCASCYGCGGTAATTCC	ACTTTCGTTCTTGATYRA
Barcoding			
	jgLCO/HCO	TITCIACIAAYCAYAARGAYATTGG	TAIACYTCIGGRTGICCRAARAAYCA
	dgLCO/HCO	GGTCAACAAATCATAAAGAYATYGG	TAAACTTCAGGGTGACCAAARAAYCA

Table S3: Chlorophyll a concentrations (mg m^3) and sea surface temperature (C) for the various sites derived from NASA's Oceancolor website (https://oceancolor.gsfc.nasa.gov/) derived from the MODIS A satellites at a 4km resolution. The two sites at Al Fahal S were combined as were the two Al Wasel sites as they were not greater than 4km apart while data for KAEC was unavailable.

Chlorophyll a (mg m^3)								
	Abu Shosha	East Fsar	Abu Shootaf	Al Fahal N	Al Fahal S	Al Wasel	Abu Madafi	Shib Nazar
Mar-13	0.4		0.6	0.4	0.6	0.4	0.3	0.2
Apr-13	0.5	1.5		0.3	0.4	0.4	0.2	0.2
May-13	0.4			0.3	0.7	0.5	0.2	0.3
Jun-13	0.2	1		0.2	0.7	0.4	0.2	0.2
Jul-13				0.2	0.7	0.5	0.2	0.2
Aug-13	0.4			0.3	0.5	0.5	0.2	0.2
Sep-13		1.2		0.3	0.6	0.6	0.3	0.3
Oct-13	0.5	1.7	1.3	0.4	0.7	0.5	0.3	0.3
Nov-13	0.5	1.5	1.2	0.4	0.6	0.5	0.4	0.3
Dec-13	0.5	1.2	1	0.5	0.7	0.7	0.4	0.4
Jan-14	0.8	1.7	1.3	0.7	0.9	0.8	0.5	0.6
Feb-14	0.8	1.5	1.2	0.6	1.2	0.9	0.5	0.5
Mar-14	0.4	1.6		0.4	0.8	0.4	0.3	0.5
Apr-14							0.4	0.4
May-14				0.2	0.5	0.4	0.2	0.2
Jun-14				0.3		0.2	0.3	0.3
Jul-14	0.2	1.9	1	0.2	1.1	0.5	0.3	0.3
Aug-14						1.4	0.3	0.2
Sep-14	0.2	1.5	0.6	0.2	0.9	0.4	0.2	0.2
Oct-14	0.4	1.6	1.1	0.4	1	0.5	0.3	0.2
Nov-14	0.4	1.4	0.9	0.5	0.8	0.4	0.3	0.3
Dec-14	0.5	1.4	1	0.5	1	0.5	0.4	0.3
Jan-15	0.4	1.3	1	0.5	0.9	0.6	0.5	0.4
Feb-15	0.8	1.5	1.1	0.9	1.1	0.7	0.6	0.4
Mar-15	0.5		1	0.4	0.7	0.5	0.6	0.3
Apr-15	0.4		0.8	0.4	0.6	0.4	0.4	0.3
May-15	0.3		0.8	0.5	0.6		0.3	0.2
Jun-15	0.3	1.4	1.1	0.4	0.7	0.4	0.3	0.3
Jul-15	0.2	1.8	1	0.3	0.9	0.4	0.3	0.2

Table S4: The number of observed OTUs in ARMS (all fractions combined) for each replicate (denoted by the A, B, C characters) analyzed.

	COI	18S
Abu.Shoosha_A	501	738
Abu.Shoosha_B	471	985
Abu.Shoosha_C	496	NA
FE_A	592	731
FE_B	650	709
FE_C	624	712
KAEC_A	647	697
KAEC_B	421	894
KAEC_C	603	709
Abu.Shootaf_A	590	NA
Abu.Shootaf_B	594	736
Abu.Shootaf_C	667	633
Al.Fahal.N_A	501	773
Al.Fahal.N_B	568	672
Al.Fahal.N_C	751	864
Al.Fahal.S_A	559	739
Al.Fahal.S_B	681	825
Al.Fahal.S_C	715	891
Al.Fahal_A	NA	685
Al.Fahal_B	995	NA
Al.Fahal_C	753	570
Al.Wasel.N_A	843	761
Al.Wasel.N_B	857	646
Al.Wasel.N_C	828	NA
Al.Wasel.S_A	486	NA
Al.Wasel.S_B	622	NA
Al.Wasel.S_C	742	690
Shib.Nazar_A	628	752
Shib.Nazar_B	542	684
Shib.Nazar_C	508	575
Abu.Madafi_A	988	881
Abu.Madafi_B	786	949
Abu.Madafi_C	926	NA

Table S5: The number of OTUs comprising each taxon for both primer sets.

		18S	COI
Alveolata			
	Apicomplexa	264	-
	Ciliophora	147	-
	Dinophyta	299	-
	Perkinsea	37	-
Amoebozoa		61	20
	Breviatea	2	-
	NAMAKO-1	1	-
	YS16Ec34	2	-
	Entamoeba	1	-
	Variosea	5	-
	Himatismenida	4	
	Stygamoebida	2	
	Discosea	11	-
	Pellita	1	-
	Tubulinea	24	-
Apusozoa			
	Apusomonadidae_Group-1	13	-
	Apusomonadidae_Group-2	1	-
	Multimonas	1	-
	Planomonadida	18	-
	Rigifilida	1	-
	Mantamonas	1	-
Arcellinida			
	Lesquereusiidae	2	
Archaeplastida			
	Charophyta	-	1
	Chlorophyta	44	14
	Glaucophyta	-	1
	Rhodophyta	128	68
	Streptophyta	2	2
Chromerida			
	Chromera	1	-
Excavata			
	Fornicata	3	-
	Preaxostyla	7	-
	Centroheliozoa	33	-
	Cryptophyceae	8	-

	Noelaerhabdaceae	2	4
	Pavlovophyceae	1	-
	Picobiliphyta	-	-
	Telonemia	1	-
	Katablepharidaceae	2	-
Opisthokonta			
	Choanoflagellatea	22	-
	Ascomycota	3	4
	Basidiomycota	5	12
	Blastocladiomycota	1	-
	Chytridiomycota	13	-
	Eccrinales	1	-
	Entomophthoromycota	1	-
	Mortierellomycotina	1	-
	Corallochytrea	1	-
	Ichthyosporea	14	-
	Nucleariidea	1	-
	Annelida	216	152
	Arthropoda	297	1403
	Brachiopoda	1	1
	Bryozoa	31	20
	Chaetognatha	1	4
	Cnidaria	48	99
	Chordata	32	173
	Ctenophora	1	-
	Echinodermata	9	28
	Entoprocta	12	4
	Gastrotricha	37	7
	Hemichordata	1	2
	Kinorhyncha	-	2
	Mollusca	69	188
	Myxozoa	1	-
	Nematoda	177	24
	Nemertea	16	12
	Placozoa	1	-
	Platyhelminthes	271	55
	Porifera	46	41
	Priapulida	-	1
	Rotifera	2	6
	Scalidophora	5	-
	Sipuncula	5	2

	Tardigrada		5
	Urochordata	26	-
	Xenacoelomorpha		15
Rhizaria			
	Acantharea	14	-
	Polycystinea	9	-
	RAD-A	16	-
	RAD-B	9	-
	RAD-C	1	-
	Cercozoa	375	6
Stramenopiles			
	Aurearenophyceae	1	-
	Bacillariophyceae	180	89
	Chrysophyceae	6	-
	Dictyophyceae	5	-
	Pelagophyceae	1	-
	Phaeophyceae	6	35
	Phaeothamniophyceae	1	-
	Pinguiophyceae	4	2
	Raphidophyceae	3	-
	Synchromophyceae	1	-
	Xanthophyceae	3	1
	Bangiophyceae	1	-
	Bicoecea	22	-
	Bicosoeca	1	
	Bigyra	1	1
	Bolidophyceae	1	-
	Hyphochytriomyceta	1	-
	Labyrinthulea	89	-
	MAST	24	-
	MOCH	3	-
	Oomycota	35	-
	Pirsonia	1	-

Table S6: Pairwise comparisons of the community structure and composition. Factors are: Pl = Placement, Se = Shelf Position and Fr = Fraction

Bray Curtis - COI

Source	df	SS	MS	Pseudo-F	P(perm)	perms	P(MC)
Shelf (Sh)	2	1.1586	0.57929	4.7131	0.001	997	**0.001**
Placement (Pl)	1	1.2079	1.2079	9.8277	0.001	997	**0.001**
Fraction (Fr)	2	2.6343	1.3171	10.716	0.001	998	**0.001**
ShxRe	2	0.8976	0.4488	3.6514	0.001	996	**0.001**
ShxFr	4	0.5693	0.14232	1.158	0.04	997	0.09
RexFr	2	0.70268	0.35134	2.8585	0.001	995	**0.001**
ShxRexFr	4	0.50987	0.12747	1.0371	0.322	991	0.36
Res	80	9.8328	0.12291				
Total	97	18.392					

Pairwise tests

Term 'ShxRe' for pairs of levels of factor 'Shelf'

Within level 'May' of factor 'Placement'

Mid ≠ Off

Mid ≠ Near

Off ≠ Near

Within level 'March' of factor 'Placement'

Mid ≠ Off

Mid ≠ Near

Off ≠ Near

Term 'ShxRe' for pairs of levels of factor 'Placement'

Within level 'Mid' of factor 'Shelf'

March ≠ May

Within level 'Off' of factor 'Shelf'

March ≠ May

Within level 'Near' of factor 'Shelf'

March ≠ May

Term 'RexFr' for pairs of levels of factor 'Placement'

Within level '106' of factor 'Fraction'

March ≠ May

Within level '500' of factor 'Fraction'

March ≠ May

Within level 'Sessile' of factor 'Fraction'

March ≠ May

Term 'RexSi' for pairs of levels of factor 'Fraction'

Within level 'May' of factor 'Placement'

106 ≠ 500

106 ≠ Sessile

500 ≠ Sessile

Within level 'March' of factor 'Placement'

106 ≠ 500

106 ≠ Sessile

500 ≠ Sessile

Bray Curtis - 18S

Source	df	SS	MS	Pseudo-F	P(perm)	perms	P(MC)
Shelf (Sh)	2	0.9133	0.45665	3.5205	0.001	997	**0.001**
Placement (Pl)	1	1.2128	1.2128	9.3502	0.001	999	**0.001**
Fraction (Fr)	2	3.4819	1.741	13.422	0.001	998	**0.001**
ShxRe	2	0.61653	0.30826	2.3766	0.001	996	**0.001**
ShxFr	4	0.60422	0.15105	1.1646	0.045	997	0.098
RexFr	2	0.98064	0.49032	3.7801	0.001	998	**0.001**
ShxRexFr	4	0.58425	0.14606	1.1261	0.081	998	0.14
Res	74	9.5985	0.12971				
Total	91	19.184					

Pairwise tests

Term 'ShxRe' for pairs of levels of factor 'Shelf'

Within level 'May' of factor 'Placement'

Mid ≠ Off

Mid ≠ Near

Off ≠ Near

Within level 'March' of factor 'Placement'

Mid ≠ Off

Mid ≠ Near

Off ≠ Near

Term 'ShxRe' for pairs of levels of factor 'Placement'

Within level 'Mid' of factor 'Shelf'

March ≠ May

Within level 'Off' of factor 'Shelf'

March ≠ May

Within level 'Near' of factor 'Shelf'

March ≠ May

Term 'RexFr' for pairs of levels of factor 'Placement'

Within level '106' of factor 'Fraction'

March ≠ May

Within level '500' of factor 'Fraction'

March ≠ May

Within level 'Sessile' of factor 'Fraction'

March ≠ May

Term 'RexSi' for pairs of levels of factor 'Fraction'

Within level 'May' of factor 'Placement'

106 ≠ 500

106 ≠ Sessile

500 ≠ Sessile

Within level 'March' of factor 'Placement'

106 ≠ 500

106 ≠ Sessile

500 ≠ Sessile

Jaccard - COI

Source	df	SS	MS	Pseudo-F	P(perm)	perms	P(MC)
Shelf (Sh)	2	1.3124	0.6562	2.9279	0.001	995	**0.001**
Placement (Pl)	1	1.0019	1.0019	4.4703	0.001	997	**0.001**
Fraction (Fr)	2	2.1129	1.0565	4.7139	0.001	997	**0.001**
ShxRe	2	1.0393	0.51963	2.3186	0.001	994	**0.001**
ShxFr	4	1.0662	0.26655	1.1893	0.001	996	**0.022**
RexFr	2	0.80141	0.40071	1.7879	0.001	998	**0.001**
ShxRexFr	4	0.96184	0.24046	1.0729	0.087	995	0.214
Res	80	17.93	0.22412				
Total	97	26.792					

Pairwise tests

Term 'ShxRe' for pairs of levels of factor 'Shelf'

Within level 'May' of factor 'Placement'

Mid ≠ Off

Mid ≠ Near

Off ≠ Near

Within level 'March' of factor 'Placement'

Mid ≠ Off

Mid ≠ Near

Off ≠ Near

Term 'ShxRe' for pairs of levels of factor 'Placement'

Within level 'Mid' of factor 'Shelf'

March ≠ May
Within level 'Off' of factor 'Shelf'
March ≠ May
Within level 'Near' of factor 'Shelf'
March ≠ May

Term 'ShxFr' for pairs of levels of factor 'Shelf'
Within level '106' of factor 'Fraction'
Mid ≠ Off
Off ≠ Near
Within level '500' of factor 'Fraction'
Mid ≠ Off
Off ≠ Near
Within level 'Sessile' of factor 'Fraction'
Mid ≠ Off
Mid ≠ Near
Off ≠ Near

Term 'ShxSi' for pairs of levels of factor 'Fraction'
Within level 'Mid' of factor 'Shelf'
106 ≠ 500
106 ≠ Sessile
500 ≠ Sessile
Within level 'Off' of factor 'Shelf'
106 ≠ Sessile
Within level 'Near' of factor 'Shelf'
106 ≠ 500
106 ≠ Sessile
500 ≠ Sessile

Term 'RexSi' for pairs of levels of factor 'Fraction'
Within level 'May' of factor 'Placement'
106 ≠ 500
106 ≠ Sessile
500 ≠ Sessile
Within level 'March' of factor 'Placement'
106 ≠ 500
106 ≠ Sessile
500 ≠ Sessile

Jaccard - 18S

Source	df	SS	MS	Pseudo-F	P(perm)	perms	P(MC)

Shelf (Sh)	2	0.90483	0.45241	2.0659	0.001	996	**0.001**
Placement (Pl)	1	1.0534	1.0534	4.8102	0.001	998	**0.001**
Fraction (Fr)	2	2.3832	1.1916	5.4415	0.001	997	**0.001**
ShxRe	2	0.79452	0.39726	1.8141	0.001	993	**0.001**
ShxFr	4	0.93193	0.23298	1.0639	0.101	995	0.249
RexFr	2	1.0778	0.53888	2.4608	0.001	993	**0.001**
ShxRexFr	4	0.92508	0.23127	1.0561	0.126	996	0.268
Res	74	16.205	0.21899				
Total	91	24.975					

Pairwise tests

Term 'ShxRe' for pairs of levels of factor 'Shelf'

Within level 'May' of factor 'Placement'

Mid ≠ Off

Mid ≠ Near

Off ≠ Near

Within level 'March' of factor 'Placement'

Mid ≠ Off

Mid ≠ Near

Off ≠ Near

Term 'ShxRe' for pairs of levels of factor 'Placement'

Within level 'Mid' of factor 'Shelf'

March ≠ May

Within level 'Off' of factor 'Shelf'

March ≠ May

Within level 'Near' of factor 'Shelf'

March ≠ May

Term 'RexFr' for pairs of levels of factor 'Placement'

Within level '106' of factor 'Fraction'

March ≠ May

Within level '500' of factor 'Fraction'

March ≠ May

Within level 'Sessile' of factor 'Fraction'

March ≠ May

Term 'RexSi' for pairs of levels of factor 'Fraction'

Within level 'May' of factor 'Placement'

106 ≠ 500

106 ≠ Sessile

500 ≠ Sessile
Within level 'March' of factor 'Placement'
106 ≠ 500
106 ≠ Sessile
500 ≠ Sessile

Table S7: The contribution of each component of beta diversity (Replacement/Turnover and Richness) and the subsequent ratio between replacement and richness. Values were separated by shelf position and the average ratio calculated for each shelf position.

	Near	
Replacement	Richness	Repl:Rich
0.5562	0.0432	12.8667
0.5747	0.0071	80.8
0.5614	0.1106	5.0769
0.5023	0.1717	2.9262
0.5018	0.1477	3.3984
0.5195	0.167	3.1096
0.5178	0.1183	4.375
0.547	0.1229	4.451
0.5747	0.0359	16
0.5237	0.1509	3.4711
0.4468	0.2139	2.0894
0.4982	0.1841	2.7059
0.4776	0.2071	2.3068
0.577	0.0755	7.64
0.5257	0.1614	3.2576
0.531	0.1191	4.4583
0.4652	0.1818	2.5584
0.5	0.1538	3.25
0.4936	0.1758	2.8079
0.5497	0.1096	5.0133
0.554	0.1283	4.3178
0.5194	0.0661	7.8621
0.5488	0.0372	14.75
0.6321	0.0581	10.8727
0.4781	0.2198	2.1754
0.6776	0.0121	56.1818
0.5635	0.0287	19.6154
0.7052	0.003	236
0.43	0.2766	1.5546
0.6359	0.0493	12.8936
0.6632	0.0238	27.913
0.4516	0.2519	1.7931
0.6863	0.0221	31.0476
0.4044	0.2787	1.4513
0.6205	0.0469	13.2273
0.4173	0.2388	1.7473

	Average	17.1102

Replacement	Mid Richness	Repl:Rich
0.6485	0.0046	142.5
0.5839	0.0817	7.1429
0.5851	0.1067	5.4831
0.6947	0.0243	28.5455
0.5641	0.1539	3.6646
0.6845	0.0346	19.8065
0.6102	0.0929	6.5714
0.5657	0.1254	4.512
0.3977	0.3261	1.2198
0.5561	0.1563	3.5583
0.477	0.2285	2.087
0.4805	0.2367	2.03
0.4821	0.2181	2.2101
0.5627	0.1267	4.4423
0.6493	0.0347	18.6875
0.5494	0.1486	3.6974
0.5913	0.0771	7.6712
0.6007	0.1095	5.4839
0.6873	0.0287	23.9231
0.5709	0.1494	3.8217
0.6902	0.0386	17.8857
0.6325	0.0873	7.2414
0.6035	0.1182	5.1074
0.4206	0.3183	1.3217
0.5671	0.1513	3.7484
0.4919	0.2227	2.2088
0.5017	0.2299	2.1825
0.5135	0.2101	2.4444
0.5583	0.1311	4.2593
0.6652	0.03	22.1429
0.5907	0.1406	4.2027
0.5656	0.1785	3.1687
0.6457	0.1005	6.4242
0.6696	0.0744	9
0.6304	0.1109	5.6852
0.7271	0.0131	55.5714
0.6869	0.0441	15.5833
0.4855	0.2496	1.9451
0.6432	0.0775	8.3023

0.5688	0.1494	3.8068
0.5475	0.161	3.4
0.5773	0.1383	4.1739
0.5145	0.2016	2.5525
0.6844	0.0444	15.4222
0.6606	0.0677	9.76
0.4607	0.0908	5.0746
0.3124	0.2809	1.112
0.5192	0.0768	6.7586
0.3669	0.2158	1.7
0.375	0.2432	1.5421
0.3319	0.4141	0.8016
0.4426	0.2606	1.6984
0.4019	0.3242	1.2398
0.439	0.3242	1.3539
0.4198	0.312	1.3456
0.6586	0.0201	32.8
0.5501	0.141	3.9008
0.4888	0.2454	1.9917
0.3638	0.1993	1.8251
0.5693	0.0113	50.2222
0.4558	0.1281	3.5575
0.4706	0.1572	2.9932
0.4016	0.343	1.171
0.5235	0.1814	2.8865
0.4546	0.2521	1.8036
0.5173	0.25	2.0692
0.4986	0.2357	2.1154
0.6095	0.1004	6.0732
0.6404	0.059	10.8519
0.5731	0.1673	3.4253
0.4272	0.201	2.125
0.5099	0.0694	7.3429
0.5803	0.034	17.0556
0.5411	0.1789	3.0246
0.7106	0.0017	415
0.6304	0.0747	8.4348
0.6567	0.0831	7.9057
0.6591	0.0623	10.5714
0.4451	0.2743	1.6226
0.5923	0.1209	4.8992
0.7325	0.0076	96.4444
0.4163	0.1419	2.9344

0.4473	0.1694	2.641
0.3874	0.3533	1.0963
0.5045	0.1927	2.6186
0.4645	0.2587	1.7958
0.4991	0.2609	1.9128
0.4821	0.2466	1.9554
0.6077	0.0909	6.6849
0.6239	0.0697	8.9524
0.5465	0.1792	3.0492
0.5348	0.0348	15.3529
0.4855	0.239	2.0318
0.612	0.0664	9.2222
0.5651	0.1379	4.0988
0.6088	0.1429	4.2614
0.5942	0.1248	4.7619
0.4782	0.2179	2.1949
0.6366	0.0591	10.7797
0.6726	0.0546	12.3279
0.5049	0.2104	2.4
0.6589	0.0338	19.4737
0.5997	0.1063	5.6406
0.6255	0.1139	5.493
0.6303	0.0935	6.7434
0.4771	0.2439	1.9563
0.629	0.0892	7.0538
0.7096	0.0235	30.2222
0.5314	0.1786	2.9752
0.5936	0.1074	5.5263
0.6142	0.0961	6.3913
0.5976	0.1177	5.0778
0.3319	0.4267	0.778
0.4728	0.2863	1.6515
0.5601	0.1831	3.0593
0.6392	0.0726	8.8
0.6288	0.0832	7.5577
0.6591	0.0607	10.8533
0.4263	0.279	1.5281
0.5846	0.1231	4.7481
0.7161	0.0094	76.3636
0.6876	0.0107	64.1429
0.6672	0.0119	56.2667
0.3726	0.3446	1.0812
0.5053	0.1959	2.5792

0.6304	0.082	7.6832
0.6578	0.0227	28.9655
0.3936	0.3477	1.1321
0.5211	0.2028	2.5702
0.6418	0.0911	7.0435
0.3812	0.3343	1.1404
0.5201	0.1841	2.8252
0.6359	0.0708	8.9767
0.4313	0.1715	2.5147
0.4077	0.2747	1.4844
0.5278	0.119	4.4333
	**Average**	**12.8319**

	Off	
Replacement	Richness	Repl:Rich
0.5956	0.1436	4.1485
0.6685	0.0418	16
0.5019	0.2729	1.8389
0.4154	0.3577	1.1614
0.4116	0.3859	1.0667
0.5991	0.1059	5.6571
0.6495	0.1357	4.7848
0.5417	0.2263	2.3934
0.5404	0.2581	2.0935
0.5208	0.238	2.1879
0.4144	0.3288	1.2604
0.4117	0.3585	1.1483
0.4436	0.1066	4.1628
0.4398	0.1491	2.95
0.5252	0.0463	11.3529
	**Average**	**4.1471**

Table S8: Details of the distance – similarity regression analysis including the intercept, slope and R^2 value. Regression analysis was undertaken using both the Bray Curtis and Jaccard dissimilarity measures for both the 18S and COI gene datasets.

COI

	Bray Curtis			
	Total	Sessile	106 - 500µm	500 - 2000µm
Intercept	0.53	0.51	0.46	0.43
Slope	-0.0011	-0.00068	-0.0015	-0.0011
R2	0.123	0.0423	0.174	0.093
	Jaccard			
	Total	Sessile	106 - 500µm	500 - 2000µm
	0.32	0.35	0.3	0.29
	-0.0011	-0.00095	-0.0015	-0.00074
	0.204	0.167	0.233	0.101

18S

	Bray Curtis			
	Total	Sessile	106 - 500µm	500 - 2000µm
Intercept	0.54	0.46	0.51	0.42
Slope	-0.0018	-0.00094	-0.0025	-0.00088
R2	0.153	0.0529	0.183	0.0383
	Jaccard			
	Total	Sessile	106 - 500µm	500 - 2000µm
	0.38	0.33	0.34	0.3
	-0.0013	-0.0007	-0.002	-0.00098
	0.166	0.0612	0.213	0.0983
